# Ochratoxin A: General Overview and Actual Molecular Status

**DOI:** 10.3390/toxins2040461

**Published:** 2010-03-29

**Authors:** André el Khoury, Ali Atoui

**Affiliations:** 1 Centre d’analyses et de recherches, Faculté des Sciences, Université Saint-Joseph, Beyrouth, Lebanon; 2 Lebanese Atomic Energy Commission-CNRS, P.O. Box 11-8281, Riad El Solh, 1107 2260 Beirut, Lebanon

**Keywords:** Ochratoxin A, toxicity, polyketide synthase gene, molecular biology, biosynthesis, detection, quantification

## Abstract

Ochratoxin A (OTA) is a mycotoxin produced by several species of *Aspergillus* and *Penicillium* fungi that structurally consists of a para-chlorophenolic group containing a dihydroisocoumarin moiety that is amide-linked to L-phenylalanine. OTA is detected worldwide in various food and feed sources. Studies show that this molecule can have several toxicological effects such as nephrotoxic, hepatotoxic, neurotoxic, teratogenic and immunotoxic. A role in the etiology of Balkan endemic nephropathy and its association to urinary tract tumors has been also proved. In this review, we will explore the general aspect of OTA: physico-chemical properties, toxicological profile, OTA producing fungi, contaminated food, regulation, legislation and analytical methods. Due to lack of sufficient information related to the molecular background, this paper will discuss in detail the recent advances in molecular biology of OTA biosynthesis, based on information and on new data about identification and characterization of ochratoxin biosynthetic genes in both *Penicillium* and *Aspergillus* species. This review will also cover the development of the molecular methods for the detection and quantification of OTA producing fungi in various foodstuffs.

## 1. Definition

**Figure 1:**
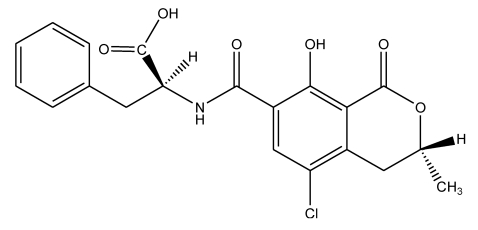
Chemical structure of ochratoxin A.

Ochratoxin A (OTA) is a mycotoxin produced by secondary metabolism of many filamentous species belonging to the genera *Aspergillus* and *Penicillium* [1,2,3,4]. Biosynthetically, it is a pentaketide derived from the dihydrocoumarins family coupled to β-phenylalanine. Its chemical name is: L-phenylalanine-*N*-[(5-chloro-3,4-dihydro-8-hydroxy-3-methyl-1-oxo-1*H*-2-benzopyrane-7-yl)carbonyl] -(*R*)-isocoumarin and its chemical structure is presented in Figure 1.

## 2. Ochratoxin A Derived Metabolites

**Figure 2:**
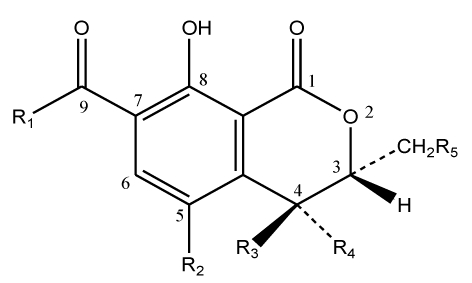
General structure of ochratoxin A metabolites.

Several metabolites related to OTA have been also identified, particularly, ochratoxin B (OTB) the dechloro analog of OTA, ochratoxin C (OTC) its ethyl ester, the isocoumaric derivative of OTA, ochratoxin α (Otα), and its dechloro analog, ochratoxin β (OTβ). Figure 2 presents the general structure common to these different metabolites and Table 1 shows the characteristic composition of each one. Recently, new OTA derived metabolites have been characterized, which include a dechlorinated ochratoxin A derivative identified by nono-ESI-IT-MS [5] and a quinone/hydroquinone metabolite showing toxicological properties [6]. In addition, it is predicted that OTA will form a benzoquinone electrophile following activation by cytochrome P450 enzymes, and radical species following activation by enzymes with peroxidase activities [7,8]. These electrophiles react preferentially with deoxyguanosine (dG) to form benzetheno adducts and C8-dG adducts, respectively [8].

**Table 1:** Characteristic composition of ochratoxin A derived metabolites.

Name	R1	R2	R3	R4	R5
Natural ochratoxins					
Ochratoxin A	Phenylalanine	Cl	H	H	H
Ochratoxin B	Phenylalanine	H	H	H	H
Ochratoxin C	Ethyl-ester, phenylalanine	Cl	H	H	H
Ochratoxin A Methyl-ester	Methyl-ester, phenylalanine	Cl	H	H	H
Ochratoxin B Methyl-ester	Methyl-ester, phenylalanine	H	H	H	H
Ochratoxin B Ethyl-ester	Ethyl-ester, phenylalanine	H	H	H	H
Ochratoxin α	OH	Cl	H	H	H
Ochratoxin β	OH	H	H	H	H
4-R-Hydroxyochratixn A	Phenylalanine	Cl	H	OH	H
4-s-Hydroxyochratoxin A	Phenylalanine	Cl	OH	H	H
10-Hydroxyochratoxin A	Phenylalanine	Cl	H	H	OH
Tyrosine analog of OTA	Tyrosine	Cl	H	H	H
Serine analog of OTA	Serine	Cl	H	H	H
Hydroxyproline analog of OTA	Hydroxyproline	Cl	H	H	H
Lysine analog of OTA	Lysine	Cl	H	H	H
Synthetic ochratoxins					
d-Ochratoxin A	d-phenylalanine	Cl	H	H	H
Ochratoxin A Ethyl amid	Ethyl amid, phenylalanine	Cl	H	H	H
O-methyl Ochratoxin A	Phenylalanine, OHCH_3 _on C-8	Cl	H	H	H

## 3. Physico-Chemicals Properties of Ochratoxin A

OTA is a weak organic acid with a pKa value of 7.1 [1,2,3] and a molar mass of 403.8 g.mol^-1^. With crystalline structure varying from colorless to white, this molecule posses an intense green fluorescence under UV light in acid medium and blue fluorescence in alkaline conditions [4].

In acid and neutral pH, OTA is soluble in polar organic solvents (alcohols, ketones, chloroform), slightly soluble in water and insoluble in petroleum ethers and saturated hydrocarbons. While in alkaline conditions, this molecule is soluble in aqueous sodium bicarbonate solution and in all alkaline solutions in general. It has a melting point of about 90 °C when crystallized from benzene as a solvate. However, non-solvated crystals of melting point 169 °C have been obtained from xylene, which are suitable for X-ray structural analysis. OTA is optically active and its spectral characteristics are shown in Table 2.

The particularity of OTA is due to its high stability. It has been shown that it possesses a resistance to acidity and high temperatures. Thus, once foodstuffs are contaminated, it is very difficult to totally remove this molecule.

In 1982, Müller [17] showed that the OTA is only partially degraded at a normal conditions of cooking. Moreover, this molecule can resist three hours of high pressure steam sterilization of 121 °C [18], and even at 250 °C its destruction is not complete [19].

Gamma irradiation (up to 7,5 Mrad) of OTA in ethanol does not cause any degradation. However, degradation is observed at low moisture level when OTA has been treated with an excess of sodium hypochlorite (NaOCl) [20]. Moreover, exposure to fluorescent light is a factor of degradation.

**Table 2:** Spectral characteristics of ochratoxin A.

Spectral	Solvents	Characteristics	References
UV-VIS	ETOH	λmax = 213nm (ε36.800)	[9]
		λmax = 332nm (ε 6.400)	
Fluorescence	ETOH 96%	λmax = 467nm	[9]
	ETOH /ABS.	λmax = 428nm	
IR	CHCl_3_	3380; 2988; 1723; 1674; 1528; 1425; 1381; 1304; 1260; 1170; 1140; 1107; 827 cm^-1^	[10]
NMR ^1^H250-MHZ	CDCl_3_	δ 12,70; δ 10,80; δ 8,55	[11,12,13,14]
		(3H); δ 7,23; δ 7,15	
		(H Aromatic); δ 4,71; δ 5,07 (CH); δ 2,78; δ 3,2 (CH_2_); δ 1,55 (CH_3_)	
MS	—	m/z 239/241	[15,16]
		m/z 255/257	
		molecular ion m/z 403	

## 4. Toxicological Profile

The toxicological status of OTA has been examined many times and was the subject of a complete monograph by the IARC (International Agency for Research on Cancer) in 1993 [21].

Following the discovery of human and animal spontaneous nephropathies, many experimental studies were carried out in order to show the implication of OTA in these diseases [22,23,24,25,26,27,28]. These studies showed that this molecule can have several effects such as nephrotoxic, hepatotoxic, neurotoxic, teratogenic and immunotoxic on several species of animals, and can cause kidney and liver tumors in mice and rats, however its toxicity varies depending on the sex, the species and the cellular type of the tested animals [29]. The genotoxic status of OTA is still controversial, due to contradictory results obtained in various microbial and mammalian tests. However, evidence of DNA-adducts formation was shown following chronic exposure of OTA to rat and sub-acute exposure to pig [30].

### 4.1. Nephrotoxicity

Nephropathy is the major toxic effect of OTA. This molecule shows to be potentially nephrotoxic in all non-ruminant mammals [31]. Epidemiological studies carried out in Denmark, Hungary, Scandinavia and Poland, showed that OTA plays an important role in the etiology of porcine nephropathy [32]. This mycotoxin was also associated with human nephropathy [33,34,35] and it is suspected to be the cause of the human fatal disease known as Balkan Endemic Nephropathy (BEN), an interstitial chronic disease affecting the south-eastern population of Europe (Croatia, Bosnia, Bulgaria and Romania) [26,33,34,35,36]. It is also considered to be the major cause of the Tunisian Nephropathy (TCIN) [37] affecting the population in Tunisia.

### 4.2. Neurotoxicity

It has been shown that the administration of OTA at gestation period in rats induced many malformations in the central nervous system [38]. In the same way, Soleas *et al.* [39] reported that OTA can be regarded as a possible cause of certain lesions as well as damage at the cerebral level. Thus, this substance seems to be highly toxic for the nervous cells and able to reach at any time the neural tissue (brain, retina) [40].

### 4.3. Teratogenicity

OTA is a potent teratogen to laboratory animals. It can cross the placenta and accumulate in fetal tissue causing various morphological anomalies. It has been reported to elicit prenatal dysmorphogenesis in rats [41,42,43,44], mice [45], hamsters [46] and chick embryos [47]. The mechanism of OTA induced teratogenesis has not been clearly defined and may involve an indirect effect through maternal action [48] and/or a direct effect on the developing conceptus [49]. Thus, the gravity of malformations depends on the route of administration and the gestative period.

### 4.4. Immunotoxicity

Under certain conditions, OTA presents a powerful immunosuppressor effect, which is observed at low or high doses [50].

Necroses of lymphoid tissues were reported indicating there high sensitivity to the OTA [50], humoral and cellular immunity affections were also described [51].

OTA seems to play a role in the inhibition of the peripherals T and B lymphocytes proliferation and stops the production of interleukin 2 (IL2) and its receptors [52]. Moreover, it blocks the activity of killer cells as well as the production of interferon [38].

The administration of OTA to many animal species causes variable effects on the osseous marrow and immunity response. Thus, this molecule is considered to be the origin of:

- Lymphopenia,
- Regression of the thymus,
- Suppression of the immunity response.

Following these results, OTA is clearly taken as an important immunosupressor agent [49].

### 4.5. Carcinogenesis

OTA is reasonably anticipated to be a human carcinogen based on sufficient evidence of carcinogenicity in experimental animals. When this molecule was administered in the diet, hepatocellular tumors (designated as well-differentiated trabecular adenomas), renal cell tumors (renal cystadenomas and solid renal-cell tumors), hepatomas (some exhibiting the trabecular structure), and hyperplastic hepatic nodules were observed in male mice [53]. 

In another study, administration of OTA in the diet induced hepatocellular carcinomas and adenomas in female mice [54]. Gavage administration of OTA to male and female rats resulted in a dose-related increase in the incidence of renal-cell adenomas and adenocarcinomas; further, metastasis of the renal-cell tumors was also observed in male and female rats. When administered by gavage, OTA increased the incidence and multiplicity of fibroadenomas of the mammary gland in female rats [21,55].

However, no adequate human studies of the relationship between exposure to OTA and human cancer have been reported. Incidence of and mortality from urothelial urinary tract tumors have been correlated with the geographical distribution of Balkan endemic nephropathy in Bulgaria and Yugoslavia [56].

## 5. Ochratoxin A Producing Fungi

OTA was isolated in 1965 from a culture of *Aspergillus ochraceus* (section *Circumdati )* [57], but subsequent studies have revealed that a variety of fungal species included in the genera *Aspergillus* and *Penicillium* are able to produce ochratoxins [57]. *A. ochraceus* has been shown to consist of two species [58,59]. The second and new species producing large amounts of OTA consistently has been described as *A. westerdijkiae*. In *Aspergillus* section *Nigri Aspergillus carbonarius* [60] is a major OTA producer. It occurs in grapes, producing OTA in grape products, including grape juice, wines and dried vine fruits [61,62] and sometimes in coffee beans [63,64]. *Aspergillus niger* aggregates have been reported as OTA producers [64,65,66,67]. The reported percentage of ochratoxigenic isolates belonging to the *A. niger* aggregate is much lower than *A. carbonarius* species [68]. * A. lacticoffeatus and A. sclerotioniger* are reported to produce OTA [69].

Others *Aspergilli* can produce OTA in large amounts, but they appear to be relatively rare. In *Aspergillus* section *Circumdati* (formerly the *Aspergillus ochraceus* group), the following species can produce OTA: *Aspergillus cretensis*, *A. flocculosus*, *A. pseudoelegans*, *A. roseoglobulosus*, *A. sclerotiorum*, *A. sulphureus* and *Neopetromyces muricatus* [59]. According to Ciegler [70] and Hesseltine, *et al.* [71] *A. melleus*, *A. ostianus*, *A. persii* and *A. petrakii* may produce trace amounts of OTA, but this has not been confirmed since publication of those papers. Strains of these species reported to produce large amounts of OTA were re-identified by Frisvad *et al.* [72]. In *Aspergillus* section *Flavi*, *Petromyces albertensis* produces OTA. 

In the genus *Penicillium*, *P*. *verrucosum* has been regarded to be the only species of the genus *Penicillium* to synthesize OTA [73]. However, it has been shown that two species of the genus *Penicillium* have this capacity, namely *P*. *verrucosum* and *P*. *nordicum*. *Penicillium verrucosum* is the major producer of OTA in stored cereals [73,74,75]. *Penicillium nordicum* [76] is the main OTA producer found in meat products such as salami and ham. 

## 6. Contaminated Foods

Cereals are the most important source of human food. The annual world crop of cereals exceeds 2,000 million tones, meaning over 160 kg per inhabitant, and the production of cereals is still growing [77,78,79]. However, an investigation carried out by Pittet [80] on a worldwide scale showed that 25% to 40% of cereals are contaminated by mycotoxins. This contamination can occur in several times (in the field and/or during storage). 

It is especially in the countries with hot and wet climatic conditions (in particular African countries, South Asia and South America) that the growth of toxigenic filamentous fungi is most favored. Thus, rice, corn, and millet: the basic foods of the populations of these countries, are often contaminated especially by aflatoxins and ochratoxins [81]. However, countries of Northern Europe, characterized by a low temperature climate, are especially contaminated by OTA where its production is due to a different species belonging to the *Penicillium* genera *(P.verrucosum, P.nordicum)*. These are species able to grow and to produce OTA at low temperatures, which can explain the fact that these countries are more contaminated with OTA producing *Penicillium* species than *Aspergillus* ones.

Thus, worldwide, cereals are considered as being the major source of OTA contamination, where 50% of human daily intake of this mycotoxin is due to the consumption of different cereals derived products [82].

Moreover, wine was recently considered as being the second source of OTA human consumption (10% to 15% of the total OTA daily intake) [83,84,85,86]. Many recent works [22,26,34,83,87,88,89,90] highlight the presence of considerable levels of this toxin in wines, musts and grape juices (up to 7 μg per liter). This occurrence of OTA in grape derived products was explained by the fact that grapes were contaminated in the vineyard from veraison onward, and sometimes even as soon as setting, by various ochratoxigenic species especially belonging to the genus *Aspergillus* section *Nigri* (*A.carbonarius*  and *A.niger aggregates*) and that OTA production increases rapidly with maturation stages [24,34,84,89,90]. Thus, the date of grape harvest would have an important effect on the OTA content in grape and its derived products.

Moreover, OTA contamination of many other raw agricultural products has been well documented; such contamination occurs in a variety of food and feed, such as coffee beans, pulses, spices, meat and cheese products [91]. OTA has also been detected in other beverages such as beer.

## 7. Regulation and Legislation

In recent years, the general concern about the potential effects of mycotoxins on the health of humans and animals has been increasing. Measures have been set up by authorities in many countries to monitor and control mycotoxin levels. Various factors play a role in decision-making processes focused on setting limits for mycotoxins. 

These include scientific factors to assess risk (such as the availability of toxicological data), food consumption data, knowledge about the level and distribution of mycotoxins in commodities, and analytical methodology. Economic factors, such as commercial, trade interests and food security issues, also have an impact. Weighing the various factors that play a role in the decision making process to establish mycotoxin tolerances is therefore of crucial importance. Despite the difficulties, mycotoxin regulations have been established in many countries during the past decades [92] and newer regulations are still being issued. 

Maximum tolerable levels and guideline levels have been established in different food and feed products, often down to the ppb or ppt level [93,94,95,96,97,98]. Such regulation has been fixed for a number of mycotoxins like aflatoxins; the trichothecenes deoxynivalenol, diacetoxyscirpenol, T-2 toxin and HT-2 toxin; the fumonisins B1, B2 and B3; agaric acid; the ergot alkaloids; ochratoxin A; patulin, phomopsins; sterigmatocystin and zearalenone [99]. Respective levels are under debate for other mycotoxins.

OTA was evaluated by the Joint FAO/WHO Expert Committee on Food Additives (JECFA) in 1991 [100] and a provisional tolerable weekly intake (PTWI) of 112 ng/kg body weight (b.w.) was established. This value was proposed according to the porcine nephropathies data basis. 

However, this molecule was re-evaluated by the JECFA in 1995 [101] and the PTWI was reconfirmed, rounding it to 100 ng/kg b.w. per week, and the request for further studies on OTA was re-iterated. 

Since its last evaluation and after the availability of several new studies on OTA effects, the JECFA recommended at its 56th meeting in February 2001 [102] that studies should be conducted to clarify the mechanism by which OTA induces nephrotoxicity and carcinogenicity and noted that studies to resolve these issues are in progress. JECFA retained the previously established PTWI of 100 ng/kg b.w. per week, pending the results of these studies.

On the other hand, the Scientific Committee on Food (SCF) expressed its opinion on OTA on 17 September 1998 that in [103]. It stated that in the light of more recent toxicological studies and exposure data, specific attention has also been given to vulnerable groups such as infants and children and groups of consumers who are exposed to higher levels of OTA than the average consumer due to their dietary habits. 

This committee proposed to reduce the OTA intake as much as possible to levels between 1.2–14 ng/kg b.w. per day.

The European Commission Regulation (EC) No. 472/2002 of 12 March 2002 [104] amended the Regulation (EC) No. 466/2001 [105] setting maximum levels for certain contaminants in foodstuffs. This regulation limits OTA contamination in unprocessed cereals, including rice and buckwheat, up to 5μg/kg. However, concerning cereal derived products, OTA contamination was fixed to 3 μg/kg. This regulation fixes also the contamination of dry grapes to a limit of 10 μg/kg. 

Moreover, the regulation (EC) No. 683/2004 of April 13 2004 [97] amended the regulation (EC) No. 466/2001 [105] including a directive limiting the OTA contamination to 0.5 μg/kg in all food preparations for babies and in diet foods for special medical purposes intended specifically for infants.

Added to that, the regulation (EC) No. 123/2005 [106] highlights the contribution of many food products such as wine, grape juices and coffee in human OTA exposure. This regulation did not modify the maximum contents established previously on cereals or grapes. However, it includes a new directive limiting OTA contamination in grape juices, wines (red, white and rosé), and must to 2 μg/L. Another new directive aiming to set limits for OTA contamination in coffee was taken; this one fixed a maximum level for OTA of up to 5 μg/kg in torrefied coffee beans and to 10 μg/kg in instant coffee.

According to the regulation (EC) No. 466/2001 [105], it is totally prohibited to mix non-conform products with conform ones in order to reduce OTA contamination levels. There was also an interdiction to use chemical treatments for OTA decontamination in products for human consumption. 

In all cases, the aim of these directives is to estimate and to minimize the presence of OTA in various foods by implementating many plans having the objective to reduce the contamination of food products by this mycotoxin. Various strategies can be adopted in order to obtain products in agreement with the international regulations. It will be a question of observing good agricultural practices, or decontaminating the end product.

## 8. Analytical Analysis of OTA

### 8.1. Chromatographical methods

The analysis of OTA in most foodstuffs is relatively straightforward and generally reliable results are obtained, as evidenced by the results from proficiency testing. Samples need to be acid- or alkali-extracted from the foodstuffs, with alkaline extraction from most matrices showing generally better recoveries [107]. 

#### 8.1.1. Thin layer chromatography (TLC)

TLC is featured in earlier AOAC methods [108,109], which use a silica gel adsorbent and an acidic solvent system. Since then, this method has been very commonly used in many laboratories around the world for identifying and quantifying the OTA in foodstuffs. The TLC consists of an OTA visual detection by its greenish fluorescence under long wave ultraviolet light, which changes to blue fluorescence after spraying the chromatographical plate with methanolic sodium bicarbonate solution or exposing it to ammonia fumes; scanning densitometric analysis may also be carried out. However, the detection limit makes this method inadequate for present day monitoring and compliance purposes. 

#### 8.1.2. Liquid chromatography (LC)

Conventionally liquid/liquid extraction and solid phase clean-up have been used prior to HPLC determination with fluorescence detection [110]. However, over the past 10 years, most laboratories have tended to move towards using immuno-affinity column clean-up because they considered it is relatively simple to carry-out and provides sample extracts generally free of interferences. 

Good methods are available for dried fruit [111], beer [112,113], coffee [114,115], dried figs [107], milk [116] and wine [22,113]. Roasted coffee tends to be the most problematic of foodstuffs to analyze for OTA and in some methods an additional clean-up step is advocated prior to the affinity column stage [117]. 

Similar methodology has been used successfully to analyze blood and urine for OTA, when this biomarker approach has been employed for exposure assessment [118]. There are CEN standards for determining OTA in cereals, roasted coffee, wine and beer with limits of quantification (LOQ) ranging from 1.3 ng/g for barley [119] and coffee [120] to 5 pg/mL for wine [22]. 

Particular attention was paid to validating a method for OTA in baby food at an LOD and LOQ of 0.05 ng/g and 0.22 ng/g respectively, the additional sensitivity being obtained by extracting larger sample sizes and enhancing fluorescence detection by using post-column ammoniation [121]. Whereas affinity column clean-up for confirmation of results is not essential, verification of results can be undertaken by methylation of OTA followed by the observation of the shift in retention time, or by LC/MS [107].

### 8.2. Immunological methods

The use of ELISA (Enzyme linked immuno sorbent assay) for OTA analysis is considered as an important and very rapid method, because it is easy to use and due to the large number of samples that can be processed at the same time (up to 90 samples for each ELISA kit in 45 minutes), and because this method do not require any clean-up procedure. ELISA methods have been applied to quantify OTA in cereals, food, feed, animals tissues and serum [122,123]. However, an important consideration with ELISA should be taken: the specificity of the antibody. Cross reactivity with related molecules can vary widely given over-estimated values.

Radio-immunoassay (RIA) for OTA has been applied to surveys of cereals, cereal products, feedstuffs, pig serum and tissues [124,125,126]. OTA has been also determined by an enzyme immunosensor with an oxygen electrode [127]. However, these methods do not appear to have been used recently.

## 9. Current Status on the Biosynthesis of Ochratoxin A

Although much information exists concerning the various toxigenic properties of OTA, unlike other important mycotoxins, not very much is known about the OTA biosynthetic pathway in any fungal species. It is widely believed that the isocoumarin group is a pentaketide formed from acetate and malonate *via* a polyketide synthesis pathway [128,129,130]. Thus, a polyketide synthase (PKS), which is considered as key enzyme, is involved in the OTA biosynthesis in a similar way of other polyketide mycotoxins such as fumonisins [131] and aflatoxins [132,133]. 

**Figure 3:**
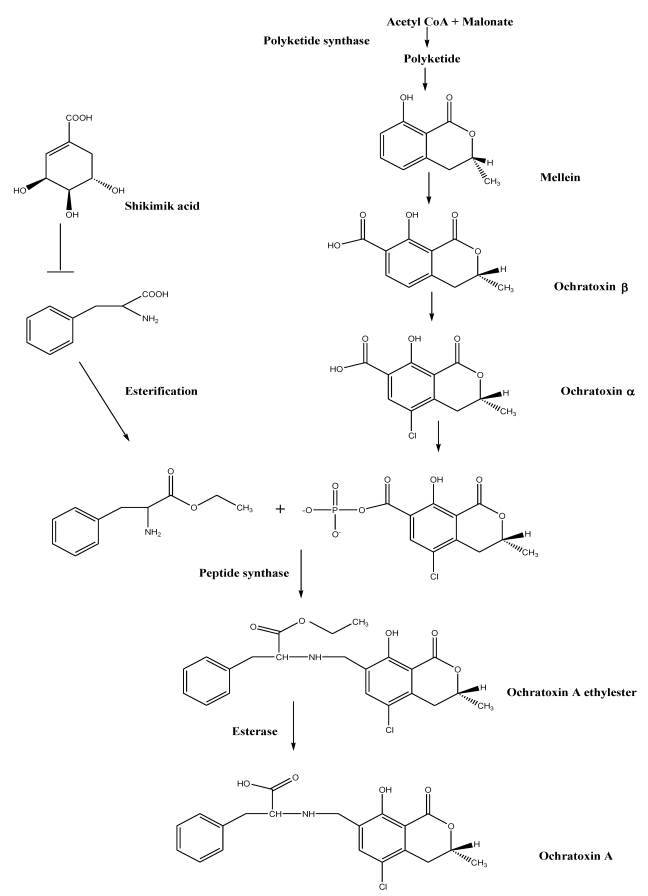
Schematic representation of the hypothetical OTA biosynthetic pathway as proposed by Huff and Hamilton, [134].

Huff and Hamilton, [134] proposed a biosynthetic pathway based on a mechanistical model according to the structure of OTA (Figure 3). The heterocyclic portion of OTA is structurally similar to mellein, a secondary metabolite produced by many OTA producing species such as *A. ochraceus*, *A. westerdijkiae* and *A. melleus*. Mellein is also produced by non ochratoxigenic species such as *Pezicula* spp. [135], *Botryosphaeria* *obtusa* [136], *Septoria* *nodorum* [137], *Phoma* *tracheiphila* [138], *Apiospora* *camptospora* [139], *Cercospora* *taiwanensis* [140], *C. scirpicola* [141], *Fusarium* *larvarum* [142], *Gyrostroma* *missouriense* [139], *Pezicula* *livida*, *Cryptosporiopsis* *malicorticis*, *Cryptosporiopsis* sp. and *Plectophomella* sp. [143].

According to Huff and Hamilton, [134], three distinct steps occur in OTA biosynthesis (Figure 3): The first part is polyketide synthesis of ochratoxin α *via* mellein involving a polyketide synthase. The second step includes acyl activation: mellein is methylated and oxidized to 7-Carboxy-Mellein (=OTβ). Chlorination by a chloroperoxidase leads to OTα. This component is then transformed to a mixed anhydride, an activation reaction using ATP. The second precursor phenylalanine is synthesized *via* the shikimic acid pathway, followed by ethyl ester activation so that it can participate in the subsequent acyl displacement reaction. In the third step, linkage of those activated precursors *via* a synthetase takes place, generating OTC, an ethyl ester of OTA: de-esterification by an esterase or transesterification is the last step in this postulated biosynthetic pathway (Figure 3). 

This schematic pathway has been dissented by Harris and Mantle [144] using labeled precursors to the growing cultures of OTA producing fungi *A. ochraceus*. Relative incorporation of labeled putative intermediates ochtatoxin α and β and mellein indicated a strong preferential role of α, moderate role of β, but found no role of mellein into OTA production.

At the molecular level, the study of genetic nature of polyketide has been facilitated by the introduction of molecular techniques such as Genomic DNA bank and cDNA bank construction; polymerase chain reaction (PCR); gene inactivation; differential display reverse transcriptase-PCR (DDRT-PCR); microarrays *etc*. PCR and subtractive PCR have been utilized to identify various PKS genes responsible for the biosynthesis of various polyketides [145,146,147,148]. Pairs of degenerated primers targeting KS domain; which is the most conserved domain among different PKSs, have been previously designed to amplify KS domain fragment from different types of PKS genes [145,146,147]. These degenerated primers were successfully used in OTA producing fungi. Five different PKS genes have been identified in *A. ochraceus* by Varga *et al*. [149] and Edwards *et al.* [129]. In addition nine different KS domains in *A. westerdijkiae* NRRL 3174 (=*A. ochraceus*) as well as five different KS domain sequences in *A. carbonarius* (2Mu134 = CBS 120167) [150] have been isolated. 

In an attempt to elucidate the molecular biosynthetic pathway of OTA, O’Callaghan *et al.* [148] have previously cloned part of the polyketide synthase (*pks*) gene (GeneBank accession number: AY272043) required for OTA biosynthesis in *A. ochraceus*, but no information was obtained concerning the presence or absence of some metabolite like the mellein. Later on, a polyketide synthase gene *otapks* PN (GeneBank accession number: AY196315), from *P. nordicum* that is essential for OTA biosynthesis was reported by Karolewiez and Geisen, [151]. Recently, Bacha *et al.* [152] reported the characterization of a PKS gene, *awks1(or aoks1 as named by the authors)*, (GeneBank Accession Number: AY583209) required for the OTA biosynthesis in *A. westerdijkiae*. Disruption of *awks1* stopped the biosynthesis of OTA, but did not affect the biosynthesis of others metabolites specially the mellein. This finding supports the results of Harris and Mantle, [144] where a mutant in which the PKS gene has been interrupted cannot synthesize OTA but still produce the mellein.

## 10. Identification of Ochratoxin A Gene Cluster

The adaptation of an organism to environmental conditions requires the cooperation of several genes that contribute to its survival. In a metabolic chain, the product of an enzymatic reaction must be taken very quickly by the following enzyme to ensure a correct speed of formation of the final product and avoid being destroyed by the possible reactions. So an enzymatic assembly in the form of complex or enzymatic cluster [153,154] is therefore a necessity. In fungi, several clusters coding for polyketides have been studied. These clusters include clusters for the biosynthesis of mycotoxins (aflatoxins, fumonisins, ergot alkaloid, paxillin), antibiotics (cephalosporin and penicillin), melanins and some pharmaceutical products (lovastatin and compactin). 

In the following paragraphs we will discuss the achievement made for the identification of OTA gene cluster in *Aspergillus* and *Penicillium* species.

### 10.1. Aspergillus species

Given that mycotoxin biosynthetic genes are often coordinately regulated [153,154] and arranged in clusters [155,157], and having established that OTA production in permissive and restrictive medium appears to be dependent on *pks* gene transcript levels expression, O’Callaghan *et al.* [158] attempted to identify other putative OTA biosynthetic genes in *A. ochraceus* by comparing their expression profiles with that of the OTA *pks* gene in permissive and restrictive medium. In their study, they focused on two putative cytochrome P450 monooxygenase genes. Both genes displayed a high degree of similarity to other monooxygenase genes encoding enzymes involved in the biosynthesis of polyketide secondary metabolites. Specifically, the p450-H11 gene is very similar to the averantin oxidoreductase gene involved in aflatoxin biosynthesis [159], while the p450-B03 gene is not only similar to the trichodiene oxygenase gene from the trichothecene biosynthetic pathway in *Gibberella zeae* [160] but also displays significant similarity to the *cyp* A gene in the aflatoxin biosynthetic pathway from *A. flavus* [161]. A strong correlation between the amount of *pks* and cytochrome P450 transcripts present and the amount of OTA accumulation was observed by the authors indicating a possible role for these genes in OTA biosynthesis. Additionally, O’Callaghan *et* al. [158] reported on the cloning of the full length pks gene and on the cloning part of a novel nonribosomal peptide synthetase (*nrps*) gene 900 bp upstream of the pks gene. Both the *pks* and *nrps* genes appear to be transcribed in the same direction but they are not in the same reading frame. Thus they appear to encode two separate proteins rather than a single hybrid type PKS-NRPS.

**Figure 4:**
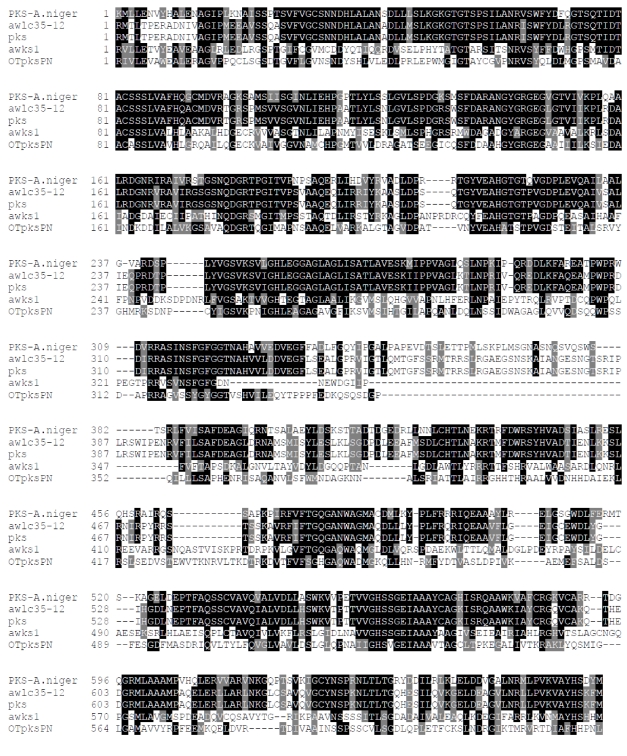
Alignment of the deduced amino acid sequence of ochratoxin A polyketide synthase gene: *awks1* (*A. westerdijkiae*, Accession Number: AY583209), *otapksPN* (*P.* *nordicum*, Accession No.: AY557343), *awlc35-12* (*A. westerdijkiae*, Accession No.: AY583208), *pks* (*A. ochraceus*, Accession No.: AY272043) and PKS- *A. niger* (hypothetical PKS gene corresponding to OTA cluster in *A. niger*, Accession No.: An15g07920).

Alignment study (Figure 4) revealed that the *pks* gene characterized by O’Callaghan *et al.* [148] shares more than 98% identity with a PKS gene *awlc35-12* (or *aolc35-12*), identified in *A. westerdijkiae* [150,162]. This finding shows that *A. ochraceus* and *A. westerdijkiae* contain a similar gene. The *pks* gene also displayed similarities of 60% to a hypothetical PKS gene corresponding to the OTA cluster in *A. niger* [163] and only 38% to a *awks1* gene required for the biosynthesis OTA in *A. westerdijkiae* [152] (Figure 4). Based on the high degree of similarities between the *awlc35-12* and *pks* genes, Bacha *et al.* [152] assumed that *awlc35-12* could be involved in OTA biosynthesis in *A. westerdijkiae*. Given that *awks1* is different from *pks*, they concluded that two PKS may be involved in the biosynthesis of OTA in *A. westerdijkiae*. To date, only four cases have been reported that involve two different fungal PKSs essential for a single polyketide: a set of two unusual type I multifunctional PKSs for the biosyntheses of lovastatin and compactin in *A. terreus* and *P. citrinum*, respectively, [164,165,166] and two PKS have been reported to be involved in the biosynthesis of zearaleone in *G. zea* [167] and T toxin in *C. heterosphorus* [168].

In *A. carbonarius*, a cDNA-AFLP differential technique was performed by Botton *et al.* [169] in two strains of *A. carbonarius*, antagonists to the ability of producing OTA, allowing the identification of 119 differentially expressed sequences putatively involved in the regulation of OTA biosynthesis. The differential conditions were achieved by growth on different minimal media, either supporting or inhibiting OTA production. A connection was pointed out between the biosynthesis of the toxin, vegetative growth and sexual/asexual developmental progression. A putative model for OTA biosynthesis regulation has been elaborated by the authors [169].

### 10.2. Penicillium species

Recently, most of the gene cluster responsible for the biosynthesis of OTA in *Penicillium* has been characterized. A 10 kb genomic DNA fragment of *P. nordicum* has been cloned [73,151,170,171], which carries three long open reading frames. One open reading frame (*otapksPN*) is a polyketide synthase, which is different from PKS genes found in *Aspergillus* species (Figure 4). The second open reading frame (*npsPN*, GeneBank accession number AY534879) has homology to non-ribosomal peptide synthetases, and the third open reading frame (*aspPN*) has homology to fungal alkaline serine proteinases. In a systematic analysis, the influence of the most important growth parameters including temperature, water activity and pH were analyzed by Real Time PCR and microarrays. Interestingly ,all analyzed external parameters resulted in a similar expression profile of the OTA biosynthesis genes. Interestingly the *otapksPN* gene is present only in *P. nordicum* but not in *P. verrucosum*, indicating genetic differences exist also between both OTA producing *Penicillium* species [73,170,171]. 

## 11. Detection of Ochratoxigenic Species by PCR

Early and rapid detection of potential OTA producing fungi is important to reduce the negative impacts of OTA. Usual identification and quantification methods of food-borne fungi require multiple steps. Morphological and physiological tests were time-consuming and often, mycological expertise was necessary [170,172]. The PCR [173] has replaced laborious and time consuming microbiological analysis by amplification of specific genomic markers rather than growing the living organism under study. Within the last 10 years PCR systems have been developed for the detection and differentiation of major species and groups of mycotoxigenic fungi. One of the most important factors in the set up of such method is the reliability of the primer set designed and the targeted DNA sequence of interest organism [173]. Recently, various pairs of PCR primers were developed to set up novel diagnostic approaches for OTA producers in the *Aspergillus* and *Penicillium* genera.

### 11.1. AFLP, RFLP, RAPD markers primers based

Pelegrinelli-Fungaro *et al.* [174] described the development of a primer pair with high specificity to *A. carbonarius*, the main OTA producer in grapes. In their study, 29 strains of *A. carbonarius*, 16 *A. tubingensis* strains and 29 strains of *A. niger* were subjected to random-amplified-polymorphic DNA (or RAPD) analysis using 471 random oligonucleotides. One particular fragment of 809 bp differentiated *A. carbonarius* from the other species. SCAR primers designed from this fragment sequence enabling differentiation of *A. carbonarius* strains from other *Aspergillus spp* by PCR. However, primers could not distinguish between toxigenic and non-toxigenic isolates of *A. carbonarius*. 

Amplified fragment length polymorphism (or AFLP) marker-based primers for *A. ochraceus* and *A. carbonarius* have also been applied [175,176,177,178]. AFLP revealed high similarity of banding patterns between the *A. ochraceus* strains [175] and also between strains of *A. carbonarius* [177]. However, both OTA producers and non-producers were scattered randomly in both species analysed. These findings were in strict contrast with results obtained by Castella *et al.* [179]. In their study, Castella *et al.* [179] classified 66 strains of *P. verrucosum* in two distinct genotypes based on AFLP and RFLP analysis. The two RAPD as well as the two AFLP groups were completely coincidental. Strains in the two groups differed in their ability to produce OTA, with group I containing mainly high producing strains, and group II containing moderate to non-producing strains. 

### 11.2. Gene-based approaches for the diagnosis of ochratoxigenic fungi

Several genes such as rRNA, β-tubulin, elongation factor 1 α and the calmodulin genes provide highly conserved as well as variable sequence regions. Based on the alignment of a partial sequence of calmodulin genes from representative strains of *A. niger* group, *A. carbonarius*, *A. japonicus* and *A. aculeatus*, Perrone *et al.* [180] detected a high degree of homology between strains assigned to *A. carbonarius* (99.98%) and also between strains of *A. japonicus* and *A. aculeatus* (99.40%). Based on variable regions, the authors found three different regions suitable for designing species-specific PCR primers. The assay was useful in screening vast numbers of isolates of black aspergilli from grapes in order to evaluate the toxigenic potential connected with this commodity. Recently, Susca *et al.* [181] developed species-specific primers based on partial calmodulin gene sequences to identify *A. carbonarius* and *A. niger* by PCR. Moreover, a primer pair targeting the conserved regions of the *A. carbonarius* calmodulin gene has also been developed by Mulè *et al.* [182], allowing only the amplification of *A. carbonarius* strains.

Morello *et al.* [183] found genetic variation between the β-tubulin gene sequences from *A. ochraceus* and *A. westerdijkiae* allowed to design a primer-pair specific to *A. westerdijkiae*. Using these primers, no PCR product from DNA of *A. ochraceus* was obtained while all isolates of *A. westerdijkiae* gave positive results.

The coding portions of many fungal 18S, 5.8S and 28S rDNA genes are highly conserved and primers to these regions have been generated [184]. The internal transcribed spacer sequences (ITS-1 and ITS-2) and IGS [185] contains hypervariable regions leading to most of the intraspecies sequence diversity [186,187,188,189]. The ITS region is amplified from the target fungus and sequenced to identify regions of DNA unique to the fungus of interest. Polymorphism within the ITS region is generally (but not always) at the level of species, rather than between isolates of the same species, making it an ideal target for the development of species-specific PCR assays. For example the species-specific primers have been designed on the basis of ITS sequence to discriminate the main species included in section Nigri: *A. japonicus*, *A. heteromorphus*, *A. ellipticus* and the two morphologically indistinguishable species of the A. niger aggregate: *A. niger* and *A. tubingensis* [190]. Other species-specific PCR-based assays to ochratoxigenic species were developed for *A. carbonarius* and *A. ochraceus* based on ITS [191]. 

Restriction digestion analysis of the ITS products was tested to assess its effectiveness as a rapid method to identify different isolates of black *Aspergillus* species from grapes [185].

Among the PCR based approaches, Accensi *et al.* [192] used a PCR-RFLP technique to distinguish *A. niger* and *A. tubingensis* isolates. The authors used the restriction enzyme *RsaI* to digest the amplified ITS region of the isolates, and observed that isolates of the *A. niger* species complex exhibit two different RFLP patterns, N and T corresponding to *A. niger* and *A. tubingensis* isolates, respectively. Other restriction enzymes has also been used for species identification recently using PCR-RFLP analysis of the ITS region [185]. The authors used *HhaI*, *NlaIII* and *RsaI* to distinguish between *A. niger*, *A. tubingensis*, *A. carbonarius* and *A. aculeatus* isolates came from grapes. Zanzotto *et al.* [193] used PCR-RFLP analysis of the ITS, IGS and β-tubulin genes to distinguish between OTA-producing and non-producing isolates of the *A. niger* aggregate.

### 11.3. Using mycotoxin biosynthetic gene for identifying of ochratoxigenic fungi

In some instances, species specific detection is not always of primary objective. It may be more relevant to determine whether the producers of particular mycotoxins are present rather than to identify exactly what the species are. This is particularly important where a mycotoxin can be produced by a number of species. Many mycotoxin biosynthetic genes are present within gene clusters, and some of these appear to have undergone horizontal transfer from one species to another and are now present in several species [153,194]. Regions of homology within mycotoxin biosynthetic gene from the different species can be used to develop primers to detect the presence of the relevant mycotoxigenic species. This strategy was successfully applied for aflatoxin producers [195,196], trichothecene-producing fungi [197,198,199], fumonisin-producing *Fusarium* species [198,199,200] and also for producers of patulin [198,201]. 

In *A. ochraceus*, the sequence of AoLC35-12 (accession nb:AY583208) was found to join a sequence encoding for acyl transferase domain of a polyketide synthase [150,162], which is involved in the OTA biosynthesis pathway [142]. Dao *et al.* [162] designed two sets of specific primers, AoLC35-12L/AoLC35-12R and AoOTAL/AoOTAR, from AoLC35-12. The set primers AoOTAL/AoOTAR specifically detected *A. ochraceus* by PCR method. Because *A. ochraceus* and *A. westerdijkiae* were only recently dismembered into two species, the primer-pairs designed until now are not specific either to *A. ochraceus* or to *A. westerdijkiae*, *i.e.*, they recognize both species. The second primer pair AoLC35-12L/AoLC35-12R was found to detect OTA producing fungi *A. carbonarius*, *A. melleus*, *A. ochraceus*, *A. sulfureus, P. verrucosum* and citrinin producing *P. citrinum* and *Monascus ruber*. 

In *A. carbonarius*, five different KS domain sequences (AcKS9, AcKS10, Ac12RL3, AcLC35-4 and AcLC35-6) were identified by Atoui *et al.* [150]. Two different PCR species specific detection systems were developed based on primers designed from AcKS10 [202] and Ac12RL3 [203] proved to be highly specific, yielding amplification only in *A. carbonarius* strains, the main OTA producer in grapes. 

In the genus *Penicillium*, a PCR method for differentiation and detection of two ochratoxingenic *Penicillium* species, *P. nordicum* and *P. verrucosum*, has been developed. It is based upon two genes of the OTA biosynthetic pathway, namely the OTA polyketide synthase gene (*otapksPN*) and a non-ribosomal peptide syntethase gene (*otanpsPN*) from *P. nordicum* [73,170]. *P. verrucosum* gives consistently only a positive reaction with the primers for the otanpsPN gene, whereas *P. nordicum* is positive for both genes. The PCR reaction is negative with all of other food related fungal species tested. This system has been used to analyze 62 *Penicillium* strains isolated from cured meat products or ripening rooms.

## 12. Application of PCR for the Detection and Quantification of OTA Producers in Contaminated Commodities

Recent advances in DNA-based techniques such as real-time PCR (RT-PCR) are providing new tools for fungal detection and quantification by detecting and quantifying their DNA. RT-PCR can be performed using different chemistries, such as SYBR^®^ Green I dye [204,205] and TaqMan^®^ [206]. Both systems have proven useful in monitoring and quantification of OTA fungal producer in many food commodities [73,170,176,182,202,203].

One of the major motivations for the development of PCR based detection systems in many publications is the prospect of using this kind of analysis to estimate OTA concentrations in sample material. One might therefore anticipate that assays based on OTA biosynthetic genes might better fit that purpose as compared to systems based on genes unrelated to their biosynthesis. By using real-time PCR, a positive correlation between OTA content and DNA quantity has been indicated for *P. nordicum* and *A. ochraceus* [164,170] and more recently, in *A. carbonarius* [182,202,203]. Such a correlation has been established with quantitative real-time PCR on mycotoxin biosynthesis genes [73] or when using primers targeted sequences of housekeeping genes [182]. 

Currently, RT- PCR quantification of *A. carbonarius* in grapes is clearly the best alternative to conventional methods in order to investigate the relation between OTA producers and OTA content. With regards to food safety, Atoui *et al.* [203] established, according to their correlation, that *A. carbonarius* DNA content has to be lower than 10 ng DNA g−1 grape berry to fulfill the maximum OTA permitted levels in the European Union (Commission regulation No. 123/2005 amending Regulation No. 67 446/2001 as regards to ochratoxin A).

All the systems described above are based on PCR with genomic target DNA as template to estimate OTA concentrations in sample materials. Given the fact that biosynthesis of most mycotoxins is a highly complex process with poorly understood regulation at the transcriptional level as well as being highly influenced by environmental factors; it is important to find correlation between gene expression and concentrations of compounds. 

A RT-PCR system based on the *otapksPN* sequence has been used to monitor growth and OTA production of *P. nordicum* in wheat [73,170]. A strong correlation between the copy numbers of the *otapksPN* gene and the colony forming units (cfu) was observed. In addition, there was a strong congruence between *otapksPN* gene expression and OTA production in wheat. It can be used for the rapid quality assessment of food products by quantitative determination of the fungal biomass. It can further be used for HACCP (hazard analysis critical control point) purposes to determine the critical control points (CCP´s) during the production chain under which otapksPN gene expression and thereby OTA production is possible. 

With the available systems of PCR-based detection and quantification of ochratoxin A described in this review, the choice of the best method depends on the goal of the study to be conducted. For example, AFLP and RAPD could be the best choice when the purpose of the study is (1) to show the polymorphism of some isolated strains belonging to the same species or (2) to discriminate between relevant OTA producer species. On the other hand, when dealing with the detection of the fungus, the best way is to conduct conventional PCR using primers designed from housekeeping genes or mycotoxin biosynthetic genes as described in sections 11.2 and 11.3. Concerning the application of the described molecular techniques in food, AFLP and RAPD show limitations such as fungal isolation and preparation of DNA of very high quality. For this reason Real time PCR technology provides an insight into the mycotoxigenic status of food sample as well as it has the power to estimate its mycotoxin content.
